# Mitral Valve Prolapse: An Innocent Bystander or Proarrhythmogenic Anomaly?

**DOI:** 10.7759/cureus.16668

**Published:** 2021-07-27

**Authors:** Raj Shukla, Dominika Zoltowska, Srinivasan Sattiraju, Jialin Su

**Affiliations:** 1 Internal Medicine, University of Florida College of Medicine – Jacksonville, Jacksonville, USA; 2 Cardiology, University of Florida College of Medicine – Jacksonville, Jacksonville, USA

**Keywords:** ventricular tachycardia (vt), sudden cardiac arrest, mitral valve prolapse, mitral annulus disjunction, transthoracic echocardiogram

## Abstract

Mitral valve prolapse (MVP) is a relatively common finding in the general population, and it is associated with ventricular tachycardia (VT) and sudden cardiac death (SCD). In this report, we present a case involving a 63-year-old male who had been previously diagnosed with MVP complicated by multiple admissions for episodes of ventricular arrhythmias.

## Introduction

The prevention of sudden cardiac arrest (SCA) and risk stratification can be challenging. Patients with known traditional risk factors classified as high-risk constitute only 30% of the total cases of SCA [1]. In fact, many causes of sudden cardiac death (SCD) are often not well understood. Therefore, to ensure better prevention of a major cardiac event, it is important to recognize non-traditional anatomic or physiologic causes that may trigger or increase the chances of cardiac arrest. As mentioned above, mitral valve prolapse (MVP) has been linked to SCA and is characterized by systolic billowing of the valve leaflets greater than 2 mm above the annular plane. It is classified as either primarily idiopathic or secondary to connective tissue or familial diseases. The pathology may present clinically with numerous nonspecific symptoms, including palpitations, chest pain, dizziness, and shortness of breath. MVP has variable correlations with other valvular or tissue pathology, including mitral regurgitation (MR), papillary muscle defects, or a rarely studied parameter known as mitral annulus disjunction (MAD). The arrhythmogenic potential and the SCA risk stratification of MVP remain an area of active investigation, and preventative intervention regarding MVP patients with underlying MAD is not yet understood.

## Case presentation

**Figure 1:**
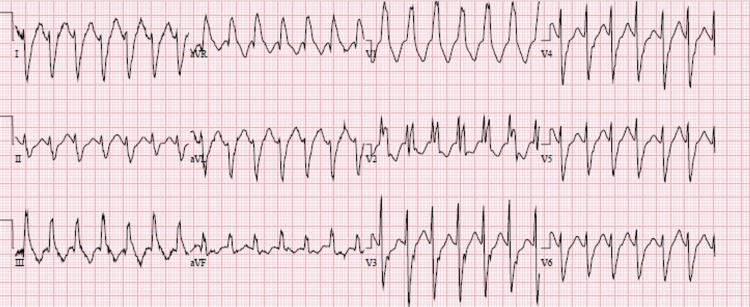
Ventricular tachycardia Ventricular tachycardia at the rate of 170 bpm, RBBB morphology with early transition in precordial leads, northwest axis. ECG suggestive of VT origin from the inferior lateral wall bpm: beats per minute; RBBB: right bundle branch block; ECG: electrocardiogram; VT: ventricular tachycardia

**Figure 2:**
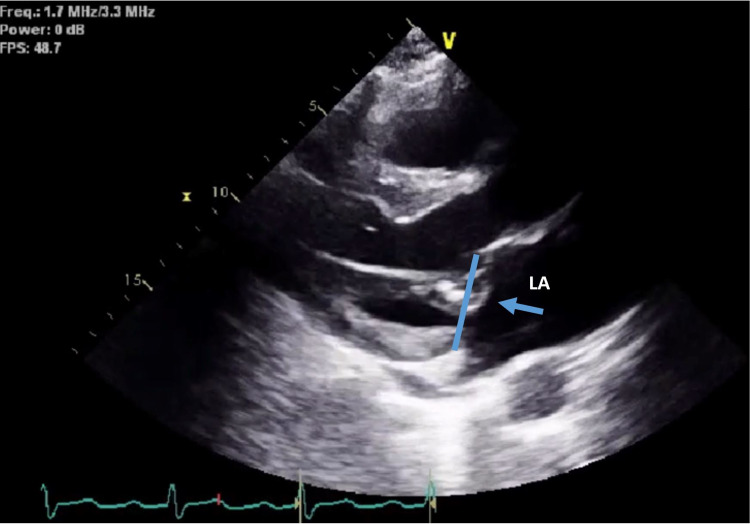
Mitral valve prolapse Significant billowing of the anterior mitral leaflets beyond the mitral annulus plane (blue arrow) LA: left atrium

We present the case of a 63-year-old man with a history of implantable cardioverter-defibrillator (ICD) insertion at the age of 42 for secondary prevention after sudden cardiac arrest (SCA) with shockable rhythm at an outside hospital; since then, he had had recurrent admissions to our facility due to episodes of ventricular tachycardia (VT) (Figure 1) despite undergoing trials of multiple anti-arrhythmic medications. He had initially been on amiodarone 400 mg a day; however, he had developed amiodarone-induced hypothyroidism and had been transitioned to sotalol 160 mg BID. The patient had no other major comorbidity or systemic symptoms, no family history of SCA, and no social history of substance abuse. Baseline ECGs showed normal sinus rhythm with normal QRS, and QT intervals. His coronary angiography revealed only mild coronary atherosclerosis (CAD) in the mid LAD (20-30% stenosis). Initial transthoracic echocardiography (TTE) was pertinent only for anterior mitral valve redundancy and prolapse (Figure 2) associated with mild MR. ICD had been implanted after his initial presentation for SCA without reversible reason, and for secondary prevention of SCA. During the two decades of follow-up, his left ventricular ejection fractions (LVEF) had remained normal; however, the MVP had become more significant years later after his initial SCA, and MR had progressed to be deemed moderate on the latest TTE (Video 1). He had undergone multiple appropriate ICD shocks for monomorphic VT (MMVT) after ICD implantation. The VT captured on 12 leads ECG was suggestive of the origin of arrhythmia from the inferolateral wall of the left ventricle. We also captured premature ventricular contractions (PVC) of at least three different morphologies. The patient could not undergo a cardiac MRI due to incompatibility with his ICD lead. He was referred for an electrophysiology study twice and ablations were performed targeting PVCs; however, he continued to have ICD shocks for VT. Ultimately, his ICD shocks decreased with mexiletine 200 mg TID.

**Video 1:** Transthoracic echocardiogram Long parasternal axis view. Anterior mitral leaflet prolapse with posteriorly directed regurgitant jet upon color Doppler interrogation

## Discussion

In cases such as ours, it can be very challenging to predict the risk of SCA prior to the initial presentation with SCA [1]. The patient's thorough workup for causes including ischemic heart disease, non-ischemic cardiomyopathy, socioeconomic potentiators, and metabolic derangements was all unrevealing. He did not have any known clinical conditions or syndromes associated with SCA (hypertrophic cardiomyopathy, arrhythmogenic right ventricular cardiomyopathy, long QT syndrome, or Brugada syndrome) either. The only positive finding noted when he initially presented for SCA with ventricular arrhythmia was anterior MVP with mild MR. ICD was implanted at the age of 42 years for secondary prevention after no reversible reason was found. During follow-up, he had PVCs with three different morphologies and had recurrent ICD shocks for MMVT. At the same time, the MVP and MR became worse. In the absence of cardiac MRI, the only positive finding in our case was evident MVP, which appeared to progressively worsen. The MVP is probably more than just an innocent bystander of the VT and SCA. It is more likely to be an arrhythmogenic anomaly in this case, given no other etiology has been identified for over more than 20 years of follow-up, and prior high-risk features of MVP have been recognized [2].

Imaging studies are crucial for the evaluation of structural heart disease or potential causes in patients presenting with VT for differential diagnosis. TTE is a well-established and easily accessible modality used for the evaluation of ejection fraction, wall motion abnormalities, and valvular disease. MVP is a relatively common condition affecting about 2% of the population [2-6]. It has been defined as at least 2-mm billowing of mitral leaflets above the mitral annulus during systole. In rare cases, MVP has been implicated as the cause of SCA. To date, some high-risk echocardiographic features predisposing patients with MVP to ventricular arrhythmias have been recognized [2], including bileaflet prolapse and severe MR. Autopsy studies have suggested that almost 99% of MVP-induced SCA have leaflet redundancy, and there was bileaflet prolapse in more than 70% of cases [3]. Moderate to severe MR appears to have an increased correlation with SCA. Peak systolic lateral mitral annulus velocity of 16 cm/s or above known as Picklehaube’s sign has also been linked to MVP with ventricular arrhythmias. With respect to mitral valve repair or replacement, some studies have shown that mitral valve repair would decrease ventricular arrhythmia, but data in the literature are mixed [7]. To date, guidelines do not recommend mitral valve surgery solely based on the coexistence of MVP and malignant arrhythmia [8]. This case only had anterior mitral prolapse with mild MR when the patient initially presented with SCA more than 20 years ago. More research is needed for the risk stratification of MVP patients. Other visible features, such as leaflet thickness, intercommissural width, anteroposterior diameter, differences in leaflet dimensions, and papillary muscle tip positions in systole vs diastole [2,4,9] have not yet been analyzed regarding their association with potential increased risk of ventricular arrhythmias and SCA.

MAD has been shown to be especially proarrhythmogenic in some studies [2,3,9]. The latest analysis has considered MAD to be relevant if there is greater than a 5-mm separation between the leaflet insertion on the atrial wall and the left ventricular basal myocardium [3]. On echocardiography, MAD is detected as an absence of visualization of myocardium between the posterior mitral valve annulus and adjacent basal segments of the left ventricular wall during systole [10]. On cardiac MRI, MAD may further be suspected in the presence of left ventricular fibrosis with gadolinium enhancement seen in the papillary muscles. The extent of disjunction can be classified by a previously established disjunction index, calculated as the product of circumferential degrees of annular involvement and the maximal long axis disjunction distance [3]. Currently, data regarding specific features of MAD is limited; however, greater longitudinal MAD distance in the posterolateral wall has been linked to an increased risk of ventricular arrhythmia. A recent study of ECG monitoring in patients with TTE findings of MVP and MAD reported ventricular arrhythmia in 43% of cases [3,9]. MAD might be an important finding in facilitating risk stratification for MVP patients.

## Conclusions

MVP can be an arrhythmogenic anomaly causing malignant ventricular arrhythmias and SCA. For patients who present with SCA, echo and possibly cardiac MRI should be performed to evaluate the mitral valve. Defibrillators should be implanted in MVP patients who have SCA for secondary prevention. To date, no guidelines or sufficient data exist regarding risk stratification in patients known to have MVP, which makes their management very difficult. Nevertheless, we believe that visualization of high-risk features related to MVP on TTE should prompt closer clinical follow-up. However, there is insufficient data to support early anti-arrhythmic medication management or ICD placement in patients with MVP for primary prevention. Therefore, we believe that further studies to develop guidelines for risk stratification of patients with evident MVP seen on TTE would be beneficial in reducing the incidence of potentially fatal incidents in this relatively benign-appearing diagnosis.
